# Clinical outcome of patients with pancreatic metastases from renal cell cancer

**DOI:** 10.1186/s12885-015-1050-2

**Published:** 2015-02-12

**Authors:** Takeshi Yuasa, Naoko Inoshita, Akio Saiura, Shinya Yamamoto, Shinji Urakami, Hitoshi Masuda, Yasuhisa Fujii, Iwao Fukui, Yuichi Ishikawa, Junji Yonese

**Affiliations:** 1 Department of Urology, Cancer Institute Hospital, Japanese Foundation for Cancer Research, Ariake, Tokyo, 135-8550 Japan; 2 Department of Pathology, Cancer Institute Hospital, Japanese Foundation for Cancer Research, Ariake, Tokyo, 135-8550 Japan; 3 Department of Gastrointestinal Surgery, Cancer Institute Hospital, Japanese Foundation for Cancer Research, Ariake, Tokyo, 135-8550 Japan

**Keywords:** Renal cell cancer, Outcome, Pancreas metastasis, Prognostic factor, Pseudocapsule

## Abstract

**Background:**

Renal cell cancer (RCC) is one of the most frequent primary sites for metastatic pancreatic tumors although metastatic tumors are rare among pancreatic malignant tumors. The purpose of this study is to disclose the characterization and treatment outcomes of pancreatic metastases from RCC.

**Methods:**

Of 262 patients with metastatic RCC treated at our hospital between 1999 and 2013, the data of 20 (7.6%) who simultaneously developed or subsequently acquired pancreatic metastases were retrospectively reviewed and statistically analyzed.

**Results:**

The median follow-up period from RCC diagnosis and pancreatic metastases was 13.4 years (inter-quartile range: IQR, 7.8–15.5 years) and 3.8 years (IQR, 2.1–5.5 years), respectively. Median duration from diagnosis of RCC to pancreatic metastasis was 7.8 years (IQR, 4.2–12.7 years). During this observation period, the estimated median overall survival (OS) time from the diagnosis of RCC to death or from pancreatic metastasis to death was not reached. The probability of patients surviving after pancreatic metastasis at 1, 3, and 5 years was 100, 87.7, and 78.9%, respectively. The estimated OS period from the diagnosis of metastases to death of the patients with pancreatic metastasis was significantly longer than that of the patients with non-pancreatic metastasis (median OS 2.7 years) (*P* < 0.0001). Surgical management for pancreatic metastasis was performed in 15 patients (75%). When the median follow-up period for these surgeries was 3.5 years (IQR, 1.9–5.2 years), the estimated median recurrence-free survival was 1.8 years. For the patients with multiple metastatic sites, molecularly targeted therapies were given to six (30%) patients. When the median follow-up period was 4.1 years (IQR, 3.0–4.4 years), no disease progression was observed.

**Conclusions:**

The pancreas is frequently the only metastatic site and metastasis typically occurs a long time after nephrectomy. The OS period of these patients is long and both surgical and medical treatment resulted in good outcomes.

## Background

Among pancreatic malignant tumors, metastatic pancreatic tumors are rare with the estimated frequency ranging from 2% to 5% [1-3]. Pancreas metastases from renal cell cancer (RCC) are also rare, with less than 2% reported in autopsy series [4,5]. In clinical practice, however, RCC is one of the most frequent primary sites for metastatic pancreatic tumors [6-8]. In addition, metastases to the pancreas from other organs are typically associated with disseminated systemic disease, but in the case of RCC, the pancreas is the only metastatic site in about half of cases and is referred to as isolated pancreatic metastasis [1-3,6-8]. Therefore, it is important to discuss the treatment strategy of pancreatic metastasis derived from RCC. Isolated pancreatic metastases from RCC are usually asymptomatic and are often detected during follow-up investigations after surgery for a primary lesion or as an incidental finding on imaging studies done for an unrelated indication [1-3,6-8]. Pancreatic metastasectomy of RCC was reported to improve survival in selected patients [6-8].

In a retrospective study, we reported that RCC was the most frequent primary site in patients with pancreatic metastases who underwent surgical resection, and in patients with pancreatic metastases from RCC, surgical resection is the treatment of choice for long-term survival (median overall survival [OS] period 45 months) [8]. However, the opportunity for surgical exploration is limited. Patients with multiple metastatic sites and widespread systemic disease at the time of diagnosis are not good candidates for resection. These patients usually undergo immunotherapy as well as molecular targeted therapy, which were introduced into the clinical practice for the treatment of metastatic RCC [9,10]. In this study, we retrospectively investigated the characterization and treatment outcomes of pancreatic metastases from patients with RCC.

## Methods

### Patients and treatment

The medical records of patients with pancreatic metastases secondary to RCC, who were treated in the Cancer Institute Hospital (Japanese Foundation for Cancer Research, Tokyo, Japan) between 1999 and 2013, were retrospectively reviewed. In all patients, pancreatic metastasis was confirmed by computed tomography and/or magnetic resonance imaging. We considered clinical and geometric factors including age, gender, Eastern Cooperative Oncology Group performance status, presence or absence of extra-pancreatic metastases, solitary or multiple pancreatic metastases, the interval from diagnosis of RCC to initial systemic therapy, surgical treatment, and systemic medical treatment, including cytokine therapy and targeted agents (sorafenib, sunitinib, axitinib, and everolimus). In this study, we applied the International Metastatic Renal Cell Carcinoma Database Consortium (IMDC) model, which stratifies patients into three risk groups (favorable: no risk factors, intermediate: one or two risk factors, and poor: three, four, five, or six risk factors) [11]. Histopathology was reviewed according to the 2004 World Health Organization classification [12]. This study was carried out in compliance with the Helsinki declaration and was approved by the institutional review board at Cancer Institute Hospital.

### Statistical analysis

Survival time was defined as the time from diagnosis of pancreatic metastasis to death or the last follow-up date. OS was estimated using the Kaplan–Meier method. The relationship between survival period and each of the variables was analyzed using the log-rank test for categorical variables. Regarding the comparison of characteristics between patients with pancreatic and non-pancreatic metastases from RCC, we used chi-square test or Student’s t-test for categorical variables. Statistical analyses were performed using the Statistical Package for Social Sciences, version 17.0 for Windows (SPSS Inc., Chicago, IL). Two-tailed *P* < 0.05 was considered significant.

## Results

### Characteristics of patients and their pancreatic metastases

**Figure 1:**
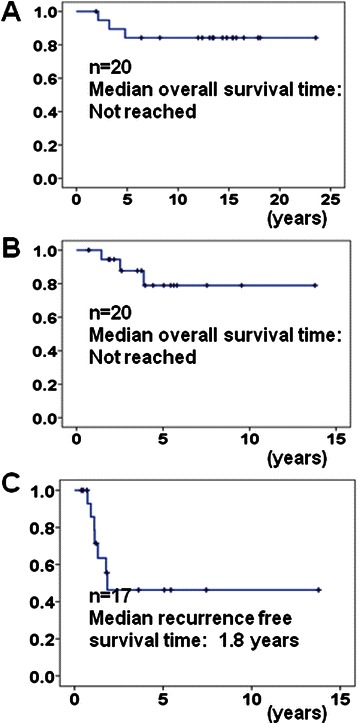
**Overall survival curves and recurrence-free survival curve of patients with pancreas metastasis from renal cell cancer.** Overall survival curve from the diagnosis of renal cell cancer **(A)** (*n* = 20). Overall survival curve from the diagnosis of pancreatic metastasis **(B)** (*n* = 20). Recurrence-free survival curve from radical surgical management of pancreatic metastases **(C)** (*n* = 17).

Of 262 RCC patients with metastases, 20 (12.0%) were diagnosed with pancreatic metastases. The median follow-up period from the diagnosis of RCC and pancreatic metastases was 13.4 years (inter-quartile range: IQR, 7.8–15.5 years) and 3.8 years (IQR, 2.1–5.5 years), respectively. Patient characteristics and demographic data are shown in Table 1. All 20 patients had undergone nephrectomy and all patients were clear cell subtype. Synchronous pancreatic metastasis was found in three patients at the time of RCC diagnosis and pancreatic metastasis was discovered in the remaining 17 patients during follow-up investigations after surgery. In patients with metachronous pancreatic metastasis, the median duration from diagnosis of RCC to pancreatic metastasis was 7.8 years (IQR, 4.2–12.7 years). During this relatively long observation period, three patients (15%) died from RCC and the estimated median OS time from the diagnosis of RCC to death or from pancreatic metastases to death was not reached (Figure 1A,B). The probability of patients surviving after pancreatic metastases at 1, 3, and 5 years was 100, 87.7, and 78.9%, respectively (Figure 1A). Among these patients, 9 of 20 (45%) had extra-pancreatic metastases at the time of diagnosis of pancreatic metastasis (Table 1). Surgical management for pancreatic metastasis was performed in 15 patients (75%). Among these patients, two patients underwent pancreas metastasectomy twice and one patient three times. One patient underwent total pancreatectomy as the second surgical procedure, whereas two patients underwent partial pancreatectomy, both as second and third surgical procedures. The latter two patients, who underwent surgery for pancreas preservation, are alive and show no signs of glucose metabolic disorder. Two patients, who had extra-pancreatic and lung metastases simultaneously, underwent surgical treatment for pancreas metastases as a palliative treatment. In the remaining 13 patients, 17 pancreas metastasectomies were performed as a radical treatment. Regarding the surgical procedure for pancreatic metastasis, two, six, two, six, and one patients underwent enucreation, pancreatoduodenectomy, segmental pancreatectomy, distal pancreatectomy, and total pancreatectomy, respectively. Average number and size of these resected pancreatic tumors was 1.8 (range, 1–6) and 20.9 mm (range, 5–53 mm), respectively. The median follow-up period for these surgeries was 3.5 years (IQR, 1.9–5.2 years) and the estimated median recurrence-free survival was 1.8 years (95% confidence interval [CI], not calculated) (Figure 1C). The probability of recurrence-free patients after metastasectomy at 1 and 5 years was 78.6% and 46.3%, respectively (Figure 1C). The 30-day perioperative morbidity rate, which included pleural effusion and wound infection, was 16%. There was no perioperative mortality. Molecularly targeted therapies were given to six (30%) patients with metastatic disease. Of these patients, one (5%) received both immune and molecularly targeted therapies. Regarding the patients who had underwent surgical resection, one patient began to receive targeted therapy due to pancreatic and lung metastases. Both sorafenib and sunitinib, sorafenib alone, and sunitinib alone were administered to one, one, and four patients, respectively. Remarkable regression was observed in all six patients (one complete responder and five partial responders). In addition, when the median follow-up period was 4.1 years (IQR, 3.0–4.4 years), no disease progression was observed in any of the six patients.

**Table 1:** **Comparison of characteristics between pancreatic and non-pancreatic metastasis from RCC (*n*** **= 262)**

Variables		Pancreatic metastasis		Non-pancreatic metastasis		*P*
Male/female	*n* (%)	12/8	(60%/40%)	184/58	(76%/24%)	0.11
Age at Dx of RCC*		59.7	(52.5–62.5)	61.7	(54.6-69.3)	0.16
Age at Dx of metastasis		66.2	(62.3–69.9)	63.6	(56.4-70.2)	0.09
Metastatic sites	*n* (%)					
	Pancreas	20	(100%)	0	(0%)	
	Lung	4	(20%)	194	(80%)	<0.0001
	Bone	0	(0%)	63	(26%)	0.009
	Kidney	5	(25%)	2	(1%)	<0.0001
	Lymph nodes	3	(15%)	77	(32%)	0.117
	Liver	3	(15%)	19	(8%)	0.268
	Adrenal gland	2	(10%)	39	(16%)	0.469
Synchronous: metachronous (*n*)		3 /17	(15%/85%)	153/89	(63%/37%)	<0.0001
Period from Dx of RCC to metastasis (years)*		7.8	(4.2–12.7)	0	(0–0.7)	<0.0001
Metastasectomy (*n*)	(Yes/No)	15/5	(75%/25%)	56/186	(23%/77%)	<0.0001
Molecular targeted therapy (*n*)	(Yes/No)	6/14	(30%/70%)	98/144	(40%/60%)	0.357
Median OS from the Dx of metastasis (years)^#^		Not reached		2.7	(1.8-3.6)	<0.0001

*Numbers represent Median (IQR), ^#^Numbers represent Median (95% CI), Dx, diagnosis; IQR, inter-quartile range; RCC, renal cell cancer; OS, overall survival; 95% CI, 95% confidence interval.

### Comparison of characteristics between pancreatic and non-pancreatic metastasis from RCC

In order to identify the characteristics of pancreatic metastasis from RCC, we compared the clinical variables between pancreatic and non-pancreatic metastasis from RCC (*n* = 262). The median follow-up period from the diagnosis of RCC and metastases of the patients with non pancreas metastasis was 2.0 years (IQR, 0.6–5.8 years) and 1.4 (IQR, 0.6–3.9 years), respectively. We summarized the characteristics of these patients and the results are shown in Table 1. The distribution of metastatic organ is apparently different between these two groups. The patients with pancreatic metastasis had less frequencies of lung and bone metastasis and more frequencies of contra-lateral kidney metastasis (Table 1). The estimated OS time from the diagnosis of metastases to death of the patients with pancreatic (median OS: not reached) was significantly longer than that of the patients with non-pancreatic metastasis (median OS: 2.7 years, 95% CI: 1.8–3.6 years) (*P* < 0.0001).

### Risk factors for poor outcome of the patients with pancreatic metastasis in univariate analysis

**Figure 2:**
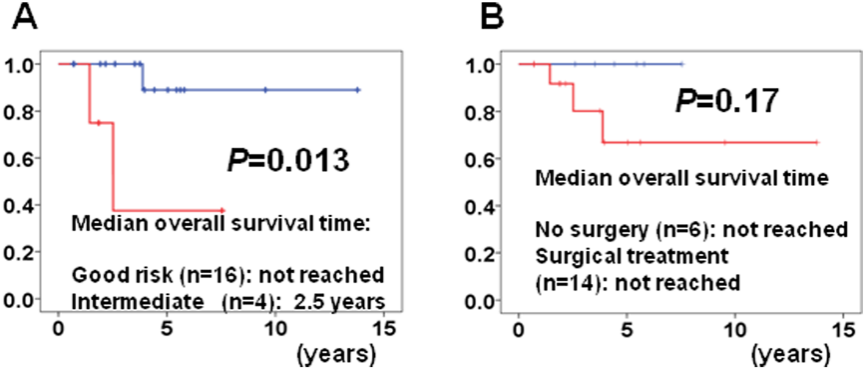
**Overall survival curves of patients with pancreatic metastasis from the diagnosis of pancreatic metastasis stratified by the International Metastatic Renal Cell Carcinoma Database Consortium (IMDC) risk scores (A), by surgical or medical treatment (B).**

Although we searched for risk factors of poor outcome using univariate analysis, we could not find any. However, when we applied the IMDC model, which stratifies patients into three risk groups (favorable: *n* = 16, intermediate: *n* = 4, and poor: *n* = 0), the estimated median OS time from the diagnosis of pancreatic metastasis was not reached for populations classified as favorable and was 2.5 years for populations classified as intermediate, which resulted in distinct separation of the OS curves (*P* = 0.013) (Figure 2A).

Next, to investigate the contribution of surgery to the outcome, we separated patients into two groups, i.e., those who received surgical treatment and those who did not. Both of median OS periods of these two groups did not reach significance (Figure 2B). In this study, the contribution of surgery to the outcome of patients with pancreatic metastases from RCC was difficult to assess.

### Pathologic findings

**Figure 3:**
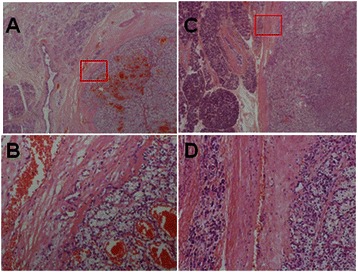
**Histopathological findings of pancreatic metastases from renal cell cancer (A–D).** High magnification images of the cells **(B and D)** captured in the red squares seen on **A** and **C**, respectively.

When we examined the pathological findings of the pancreatic metastases, which were resected by pancreatic metastasectomy, we observed a distinctive feature of RCC-derived metastatic pancreatic tumors, a fibrous pseudocapsule (Figure 3A–D). This feature is not normally observed in metastases from other organs. There might be a distinct histological difference between RCC-derived metastatic regions and those from other organs.

## Discussion

Our study demonstrated that patients with pancreatic metastases from RCC have relatively longer survival times than patients with metastatic RCC. Recent reviews show that the 5-year OS rate in patients who have received surgical treatment for pancreatic RCC metastases was 66–78% [7,13]. The reason why better outcomes are achieved in patients with pancreatic RCC metastases than in patients with metastasis to other sites is unknown. From our study and those of others, we suggest that the following factors may explain why patients with pancreatic RCC metastases had better OS: (a) late recurrence, which is a good prognostic factor in the IMDC score; (b) few of the patients were categorized with a poor prognosis using the IMDC score; (c) efficient surgical and medical treatments; (d) high population of isolated RCC metastatic disease to the pancreas; and (e) presence of a pseudocapsule in the pathological analysis (Figure 3A–D).

In the cytokine era, prognostic factors that predict the outcome in patients with metastatic RCC were defined by Motzer et al. at Memorial Sloan Kettering Cancer Center (MSKCC), which resulted in the development of an MSKCC score [14]. In the era of molecular targeted therapy, several studies have investigated clinical prognostic factors [14-17]. Among these, the score developed by Heng et al. (IMDC score) is the most popular and is widely used in clinical practice [11]. Both studies demonstrated that a poor prognostic factor was a time of less than one year from diagnosis to treatment. In addition, we recently reported that these prognostic scores were associated with prognosis among Japanese patients with metastatic RCC [18,19]. Very recently, the IMDC study also demonstrated that late relapses had significantly longer progression-free survival (*P* = 0.005) and OS (*P* = 0.004) [20]. These studies and the current study suggest that late recurrence, which is also one of the characteristics of pancreas metastasis from RCC, is an important factor for long- term survival.

When the median follow-up period for the patients treated with the targeted agents was 4.1 years, no progression was observed in all six patients. However, patient number was small, so the absence of disease progression should be confirmed in future large scale studies. As the efficacy of the targeted agent was excellent in our study, the contribution of surgery to the outcome of patients with pancreatic metastases from RCC was difficult to assess (Figure 2B). In a recent review, the estimated median OS period and 5-years OS rate of 112 patients with pancreatic metastases from RCC after surgical treatment was 8.75 years (range 0.6 months to 18.5 years) and 66%, respectively [12]. Similarly, in a recent single-center retrospective study, the survival of 23 patients undergoing surgery was compared to that of 13 non-surgically treated patients. The actual 5-year OS rates and median disease-free survival were 88% and 44 months in the surgical group and 47% and 27 months in the non-surgical group, respectively (*P* = 0.02) [7]. In our study, 2-and 5-year OS rates of the surgically treated patients were 80% and 72%, respectively. In addition, 1- and 5-year recurrence-free survival rates after metastasectomy were 78.6% and 46.3%, respectively (Figure 1C). The median recurrence-free survival time of patients who were not administered targeted agents was 1.8 years, which was better than the progression-free survival time of patients who were not administered targeted agents [9,10,21], although seven of 17 cases recurred after surgical treatment. Although we consider that surgical resection should not be the only therapeutic tool against pancreatic metastases from RCC, surgery is still an important treatment modality even in this era of targeted therapy. We believe that a combination of various treatment approaches during the different stages of metastatic RCC could result in synergistic antitumor activity.

A high proportion of patients did not have metastases other than those to the pancreas; this is referred to as isolated pancreatic metastasis. In our study, 45% of patients did not have other metastatic lesions, which is similar to the findings of other studies [1-3,6,7]. As there was no difference between the survival period with or without other metastases, the contribution of these metastases to the outcomes of the patients is difficult to assess. A relatively improved outcome may reflect a special non-aggressive characteristic of pancreatic metastasis from RCC. In addition, we cannot predict the spontaneous regression, especially in asymptomatic patients with a long disease free interval (>5 years). These patients might possibly survive anyway without surgery and/or targeted agents.

We investigated the histological features of RCC pancreatic metastasis, as tumor non-aggressiveness could be a pathological feature. In the current study, a pseudocapsule, which consists of fibrous tissue between the tumor and the pancreatic parenchyma, was observed in all of the surgically resected RCC pancreatic metastases. Some studies of liver metastasis have indicated that a pseudocapsule is a favorable prognosis factor after resection [22-25]. Okano et al. and Iguchi et al. demonstrated that a pseudocapsule has a beneficial effect on patients and increases recurrence-free survival of patients with colorectal cancer after hepatic resection [22,23]. Okano et al. and Garancini et al. demonstrated that a pseudocapsule around liver metastases from gastric cancer is a favorable prognostic factor and is closely associated with patient survival [24,25]. They speculated that the formation of a pseudocapsule could constitute a protective immunoinflammatory reaction against the metastatic nodule, and that the host defense reaction created a wall to inhibit cancer cell diffusion. Therefore, pseudocapsules may be considered as beneficial in patients with pancreatic metastases from RCC.

## Conclusion

We retrospectively investigated the clinical outcome of patients with pancreatic metastases from RCC and determined the characteristics of these metastases. The pancreas was frequently the only metastatic site and metastasis typically occurred a long time after nephrectomy. The OS period of these patients is long and both surgical and medical treatment demonstrated good efficacy. The decision making as well as surgical indication for patients with the metastatic lesion is always difficult issue. Currently we are giving all the information demonstrated here and asking the patient’s preference “surgery” or “targeted therapy”.

## Abbreviations

IMDC: International metastatic renal cell carcinoma database consortium
RCC: Renal cell cancer
MSKCC: Memorial Sloan Kettering Cancer Center
